# Assessment of Mathematics Difficulties for Second and Third Graders: Cognitive and Psychological Parameters

**DOI:** 10.3390/bs9070076

**Published:** 2019-07-12

**Authors:** Marios A. Pappas, Fotini Polychroni, Athanasios S. Drigas

**Affiliations:** 1 Department of Psychology, National and Kapodistrian University of Athens, 157 84 Athens, Greece; 2 Net Media Lab-Mind & Brain R&D, I.I.T., N.C.S.R. ‘Demokritos’, 153 41 Agia Paraskevi, Greece

**Keywords:** mathematical achievement, math difficulties, cognitive skills, working memory, math anxiety

## Abstract

Mathematical achievement during the first years of primary school seems to be a reliable predictor of students’ later performance. In addition, cognitive, metacognitive, and psychological parameters are considered to be factors related to mathematical achievement. However, in the Greek educational system, there is a shortage of valid and reliable tools for the assessment of mathematics difficulties and as a consequence, identification of children with these difficulties does not take place before the last years of primary school. This study aims to investigate the relationship between working memory, sustained attention, executive functions, and math anxiety with mathematical achievement in 2nd and 3rd graders. The design of the study was based on the parameters of mathematics difficulties, as they arise from the literature review. Ninety-one Year 2 and Year 3 primary school students (mean age 8.06 years) from three public schools situated in Attica, Greece participated in the study. The students completed three different scales including educational, cognitive, and psychological tasks. Results showed that mathematical skills were significantly correlated with sustained attention, inductive reasoning, math anxiety, and working memory. Moreover, mental arithmetic ability, sustained attention, and working memory predicted mathematical achievement of second and third graders. The study’s outcomes verify that sustained attention, inductive reasoning, working memory, and math anxiety are correlated with young students’ mathematical performance. The implications of the results for the development of an assessment tool for early detection of mathematics difficulties will be discussed.

## 1. Introduction

Early math skills are considered a strong predictor of students’ later mathematical performance [1] as well as of general subsequent academic performance [2]. Difficulties in mathematics could be attributed to external factors that are related to social problems, problems arising from school such as poorly structured curriculum or poor teaching, as well as internal factors, such as learning disabilities. As primary school students get older, their number line estimation ability, as well as their general mathematical performance, tend to grow [3], however this does not seem to be the same for all children. A critical factor for students’ attitudes towards mathematics is their arithmetic problem-solving ability during the first years of primary school, in which most students struggle [4]. In addition, inductive reasoning seems to be significantly important in the field of mathematics and especially in problem solving [5]. There is evidence to support the idea that there are cognitive abilities affecting mathematical achievement in primary school, such as working memory and attention [6,7,8,9,10], as well as executive functions such as inhibition, switching, and updating [11,12,13,14]. Working memory and executive functions are positively correlated with mental arithmetic skills, i.e., solving arithmetic problems without paper and pencil, especially for students with high levels of math anxiety [15]. Cognitive processes are involved even in basic arithmetic operations, like the carry and borrow effect in multidigit addition and subtraction [16]. In addition, math anxiety in early primary school years has negative effects on the academic, as well as on the social life, of children [17]. Recent studies indicate that as math anxiety increases, math achievement decreases [18].

General cognitive skills, as well as domain-specific components, such as quantity/number comparison or estimation (known as numerosity) and number line estimation could predict early mathematical skills [19,20]. In addition, verbal skills, executive functions, family environment, and informal math abilities, could be considered significant predictors of primary school students’ mathematical achievement [21]. Cognitive, metacognitive, and psychological parameters of mathematical achievement should be studied for the development of valid and reliable assessment tools for mathematics difficulties.

This study investigates the cognitive, psychological, and metacognitive variables that are linked to mathematical achievement in primary-school students of second and third grade. In particular, the present study aims to examine the correlation of working memory, math anxiety, and sustained attention with mathematical achievement. Another aim of the study is to investigate the role of executive function, such as inductive reasoning and problem solving in mathematical achievement. In addition, this study investigates differences between second and third graders, as well as gender-based differences in cognitive and psychological measures. Final aim was to design a curriculum-based assessment test (mathematical achievement test) for the detection of mathematics difficulties during second and third grade of primary school. We hypothesized that cognitive (sustained attention, working memory, and mental arithmetic ability) and metacognitive skills (inductive reasoning) are positively correlated with mathematical achievement; students with higher levels of math anxiety will have lower performance on mathematical tasks; cognitive and metacognitive skills of second and third graders will predict mathematical achievement; as well as that performance on mathematical and cognitive tasks will differ as a function of school year. Finally, we hypothesized that the mathematical achievement test can be used as a reliable curriculum-based assessment tool for mathematics difficulties.

## 2. Literature Review

Μathematics difficulties are associated with specific cognitive processes, such as working memory, attention regulation, and information retrieval deficits [22]. Given that there is a variety of terms used in the literature in order to describe children with difficulties in mathematics, we use the term *mathematics difficulties* (MD), as this term describes children with or without diagnosis for learning disabilities [23,24]. Other studies describe children with MD as those who perform at or below the 35th percentile [25,26]. Children with MD, especially those with difficulties in arithmetic operations, are characterized by a weak understanding of numerical magnitudes [27], i.e., mental magnitudes for number representations [28]. This extends to difficulty in proportional reasoning ability, as students with MD face difficulties in understanding ratios and proportional relationships [29]. In addition, second graders with MD face difficulty when they are asked to do approximate spatial representations of large quantities on the number line [30]. Number line estimation tasks are positively correlated with magnitude understanding and are considered indicators of students’ performance on upper level mathematics courses [31]. Given that, the number line should be used as a representational tool for fraction understanding. Primary school teachers need reliable assessment and screening tools in order to identify accurately young children at risk of mathematics difficulties and thus provide individual instruction tailored to their specific needs [32]. There is evidence of gender differences in strategy use, accuracy, and confidence on mathematics [33]. Past research shows that boys perform better than girls in problem solving, while girls are better in arithmetic operations [34]. Better performance of male students compared to their female peers on problem solving can partially explain their advantage in mathematical literacy [35].

Working memory, the ability to temporarily store and manage data during a task, is considered to be significantly correlated with performance in mathematics [36,37,38]. In particular, central executive system of working memory appears to be related to various mediating skills, such as computational ability and inhibition and naming speed [39]. Differences in mathematical performance between typically developing children and children with mathematical disabilities are even more intense in visuospatial working memory tasks [40]. Problem solving, as well as multi-digit arithmetic operations (addition, subtraction, multiplication, division), are the mathematical skills that seem to have the strongest correlation with working memory [41].

Sustained attention, i.e., the ability of an individual to maintain attention on specific stimuli [42] seems to be correlated with mathematical performance [43] and especially with number recognition and comparison, mental number line, arithmetic operations, and times-table [44]. There is also evidence for the relationship between sustained attention and various cognitive abilities, as well as with children’s school readiness [45]. The ability of pre-school children (3 to 6 years old) to select and maintain attention, could be a predictor of basic numeracy [46]. Increased levels of signal detections, decreased levels of false alarms, as well as reaction time are considered to be the crucial measures of sustained attention [47].

Inductive reasoning, as a higher-order executive function [48], is considered a significantly important factor in the field of mathematics and especially in problem solving [5]. Function-finding (or pattern recognition), meant as the ability to recognize relationships between a given set of numbers, is representative of inductive reasoning [49]. The literature review indicated a classification of seven stages of inductive reasoning: Observation of particular cases, organization of particular cases, search and prediction of patterns, conjecture formulation, conjecture validation, conjecture generalization, and general conjectures justification [50]. Inductive reasoning is a good predictor for students’ academic performance [51] and its development could positively affect a broad range of their school activities [52]. Furthermore, training with inductive reasoning tasks could positively affect children’s fluid intelligence [53].

Math anxiety, defined as the feeling of tension when dealing with mathematics, can explain the variance in math performance during elementary school years [54]. Math experience in early childhood is important for the development of math anxiety and, thus, math achievement of first and second graders could be used as a reliable predictor of math anxiety at this age [55]. In addition, high levels of math anxiety are linked with various cognitive skills such as working memory [56]. Research findings indicate that the effect of math anxiety seems to increase from Grade 2 to Grade 3 [57]. Primary-school students with high levels of math anxiety have poor mathematical performance, especially those who have also low working memory capacity [58]. In addition, Ramirez, Gunderson, Levine, and Beilock (2013) indicate that there is a negative correlation between math anxiety and the use of advanced solving strategies for first and second graders [59]. These results also supported by the study of Wu, Amin, Barth, Malcarne, and Menon (2012) in second and third graders [60]. Finally, there is evidence that female primary school students have higher levels of math anxiety compared to male students [61].

The literature review indicates the need for early identification of students with mathematics difficulties in the first primary grades [62,63,64], so that appropriate intervention can be applied. As part of broader research for the development of a reliable assessment tool for early identification of students with mathematics difficulties, this pilot study investigates the cognitive and psychological parameters of mathematical achievement in the first primary grades. In this study, firstly, we aim to investigate the relationship between mathematical achievement in early childhood and mental arithmetic ability, basic cognitive processes, and math anxiety. Secondly, we aim to identify the cognitive abilities that predict later mathematical performance. Based on the findings, we analyze the principles of cognitive-based assessment of mathematical difficulties for second and third graders, in order to propose a reliable tool for early identification of math difficulties.

## 3. Method

### 3.1. Sample

Participants were 93 primary-school students, attending the 2nd and 3rd grade, from three different public schools in Attica, Greece. Two of the participants were excluded from the analysis, due to an assessment of the autism spectrum disorder. Thus, data from 91 students (42 boys and 49 girls) were used for the analysis. Convenience sampling was used for data collection. The mean age of students was 8.06 years with standard deviation 6.82 months (minimum age = 7.16 years, maximum age = 9.16 years). There were 53 second-grade students (58.2%) and 38 third-grade students (41.8%). Moreover, 6 primary school teachers participated in the study who completed the working memory scale for each student.

### 3.2. Materials

#### 3.2.1. Mathematical Achievement

For the assessment of participants’ mathematical achievement, we designed and administered the mathematical achievement test, a curriculum-based assessment test, for 2nd and 3rd grade primary-school students. This paper and pencil test is administered individually and consists of 32 questions divided into 8 domains of 4 questions each, i.e., counting (counting of specific objects on a given picture), addition and subtraction (2-digit and 3-digit integers), multiplication (1-digit and 2-digit integers), division (1-digit and 2-digit integers), sequences (finding the missing term of an arithmetic sequence), number line estimation (representation of numerical values on a line), and problem solving (word problems with basic math operations), which should be answered within 15 min. Cronbach’s Alpha for the mathematical achievement test was 0.784.

#### 3.2.2. Sustained Attention

In order to assess sustained attention, we used the visual sustained attention task from the standardized test battery ‘Test of Attention for Elementary School Students’ [65]. This particular subtest evaluates the accuracy, as well as the speed at which a student locates specific shapes, in a dense arrangement of various shapes. The sustained attention test is administered individually and lasts maximum 3 min. For this tool, it was not possible to calculate an internal consistency index, as only the overall performance of the student is available.

#### 3.2.3. Working Memory

Working memory was assessed with the Greek version of the working memory rating scale [66]. This teacher rating scale asks the teacher to use his/her experiences with the student, in order to give information about the weaknesses and strengths of each student. The scale consists of 20 short questions, which describe behaviors of children with working memory deficits. The teacher grades whether each behavior is representative for the student, on a scale from 0 (never) to 3 (very often). Cronbach alpha for the working memory scale was 0.707.

#### 3.2.4. Inductive Reasoning

Inductive reasoning was assessed with the tasks ‘Nonverbal Logical Sequences’ (stories and geometric concepts), from the standardized test of executive function for elementary school students [65]. In these individually administered tasks, a sequence of 4 pictures (stories in the first task and geometric shapes in the second) is presented to the students. Then, they are asked to indicate the picture that completes the sequence from 4 alternative pictures. The administration discontinues after three consecutive failures. Cronbach’s alpha for the first task (stories) was 0.512, and for the second task (geometric shapes) was 0.750.

#### 3.2.5. Mental Arithmetic

In order to assess numerical accuracy, reasoning, and mental arithmetic skills, we used the arithmetic subtest of Wechsler intelligence test for children-III (Greek standardization), third edition [67]. The test consists of 24 arithmetic problems, which participants are asked to solve mentally, without using paper and pencil. The administration discontinues after three consecutive failures. The reliability index Cronbach’s alpha was 0.702.

#### 3.2.6. Math Anxiety

In order to assess participants’ math anxiety levels, we translated a modified version of the abbreviated math anxiety scale [68,69]. Two native Greek speakers fluent in English were involved in the translation. The translation process for the anxiety scale was conducted by applying the forward and back translation method [70]. Students were asked nine questions regarding their anxiety levels during engagement in mathematics. Given the young age of the students, the questionnaire was administered individually as a structured interview. To facilitate students to rate their anxiety levels during situations involving mathematics, they were asked to indicate an emotion icon from a given card with 5 icons (1 = low anxiety to 5 = high anxiety). Cronbach’s alpha for this particular test was 0.69.

### 3.3. Procedure

Data collection took place between April and June 2018. The maximum working time for each student was 45 min. Data were anonymous, as the information from the participants did not reveal their identity in any way. Ethical agreement and consent for access to schools was provided by institutional review board procedures (Departmental Ethics Committee, 20/12/2017, Law 4485/2007). Informed consent of students’ parents was ensured. After the completion of data collection, all valid data were used for the statistical analysis.

## 4. Results

For the analysis of the collected data, IBM’s SPSS was used. For the internal reliability of each scale administered, Cronbach’s alpha was measured. The structural validity of the mathematics assessment test was guaranteed using Pearson’s correlation coefficient for the scale’s questions. One-way analysis of variance (ANOVA) was used to compare means between independent groups. Finally, stepwise multiple regression analysis was performed in order to evaluate possible predictors of mathematical achievement.

### 4.1. Mathematical Achievement, Cognitive Skills, and Math Anxiety

In order to investigate the relationship between cognitive factors (sustained attention, working memory, mental arithmetic ability), metacognitive (inductive reasoning), psychological factors (math anxiety), and mathematical achievement, we calculated Pearson’s correlation coefficients for sustained attention, inductive reasoning (stories and geometry concepts), mental arithmetic ability, math anxiety, working memory, and mathematical achievement scores. The analysis (Table 1) revealed moderate positive correlations between mathematical achievement and mental arithmetic ability (r=0.542,  p<0.001) Mental arithmetic ability is mainly correlated with division (r=0.486,  p<0.001), multiplication (r=0.375,  p<0.001), and pattern recognition (r=0.375, p<0.001). In addition, a low positive correlation was found between mathematical achievement and sustained attention (r=0.368, p<0.001), as well as with inductive reasoning including geometric shapes (r=0.309, p=0.003). Moreover, there was a low negative correlation between mathematical achievement and working memory deficits (r=−0.420, p<0.001), as well as between mathematical achievement and math anxiety (r=−0.263,  p=0.012). Higher levels of math anxiety (as indicated by the abbreviated math anxiety scale) were negatively correlated with division (r=−0.255, p=0.015), pattern recognition (r=−0.238, p=0.023), and number line estimation task (r=−0.237, p=0.023). Finally, working memory deficits as rated by teachers were mainly correlated with basic arithmetic operations and specifically division (r=−0.410, p<0.001), multiplication (r=−0.286, p=0.006), and subtraction (r=−0.283, p=0.007).

A stepwise multiple regression analysis as was performed with mental arithmetic as Step 1, sustained attention as Step 2, and working memory as Step 3 with mathematical performance as a criterion variable (Table 2). As shown above, all predictor variables were significantly correlated with the criterion variable. Mental arithmetic ability is the first variable which accounts for the largest amount of variance in mathematical skills (29%). The next predictive variables are sustained attention (9%) and working memory (5%). In total, the three selected significant predictive variables predicted 43% of the overall variance.

### 4.2. Differences Between Second and Third Graders

In this section, descriptive statistics for mathematical and cognitive measures, in terms of school grade (Table 3), are presented. One-way analysis of variance revealed significant differences in the means of mathematical achievement ($F\left(1,89\right)=23.360,\;p<0.001,\;\eta^{2}=0.208)$ and specifically in counting ($F\left(1,89\right)=18.865,\;p<0.001,\;\eta^{2}=0.175)$, subtraction ($F\left(1,89\right)=31.879,\;p<0.001,\;\eta^{2}=0.264)$, multiplication ($F\left(1,89\right)=23.065,\;p<0.001,\;\eta^{2}=0.206)$, division ($F\left(1,89\right)=9.255,\;p=0.003,\;\eta^{2}=0.094)$, and pattern recognition ($F\left(1,89\right)=4.154,\;p=.045,\;\eta^{2}=0.045)$ between second and third graders. In terms of their cognitive skills, second and third graders had statistically significant mean differences in inductive reasoning ($F\left(1,89\right)=8.389,\;p=0.005,\;\eta^{2}=0.090)$ and math anxiety ($F\left(1,89\right)=4.711,\;p=0.033,\;\eta^{2}=0.050).$ Results did not show any significant gender-based differences in mathematical or cognitive skills.

### 4.3. Gender-Based Differences

One-way analysis of variance was applied in order to find gender-based differences between male and female participants in their mathematical and cognitive skills (Table 4). Analysis revealed statistically significant difference in pattern recognition $\left(F\left(1,89\right)=5.276,\;p=0.001,\;\eta^{2}=0.116\right)$ and problem solving tasks $\left(F\left(1,89\right)=5.523,\;p=0.021,\;\eta^{2}=0.058\right)$. In both categories, boys had significantly better scores than girls.

### 4.4. Validity and Reliability of the Mathematical Achievement Test

In order to evaluate the structural validity of the mathematical achievement test, correlations were calculated between the eight tasks of the test (Table 1). There were low and moderate positive correlations between the tasks, ranging from 0.161 to 0.702, excluding the counting task. In addition, Cronbach’s alpha reliability for the eight items of the test was 0.784, indicating fairly good levels of internal consistency. It should be noted that the value of Cronbach’s alpha, if the task counting was deleted, would be 0.80. In addition, the correlation coefficient (r=0.542) between mathematical achievement (mathematical achievement test) and mental arithmetic ability (WISC A\arithmetic) indicates the external validity of the test.

## 5. Discussion

Recent studies reveal that mathematical achievement during the early years of primary school is correlated to various cognitive aspects. The aim of the present study was to investigate the cognitive, psychological, and metacognitive variables that seem to affect or predict mathematical achievement in Year 2 and Year 3 primary school students.

The results of our study indicated that mathematical achievement of second and third graders is positively correlated with students’ mental arithmetic ability, sustained attention, and working memory. These results are consistent with the findings of extant literature in the field of mathematical cognition [71,72]. Furthermore, as revealed by the multiple regression analysis, mental arithmetic ability, sustained attention, and working memory could be used as predictors for an important portion of the overall variance of mathematical achievement. In addition, the results supported previous findings, indicating that students with working memory deficits have decreased mental arithmetic ability [73]. Another study also indicated that participants with working memory deficits, scored lower on the inductive reasoning scale [74].

Furthermore, results of this study revealed that second and third graders with higher levels of math anxiety scored lower on mathematical achievement tasks and especially in division, pattern recognition, and number line estimation. Finally, results showed that there were no statistically significant differences between boys and girls in terms of math anxiety levels. This is consistent with previous literature [75], however there are several studies indicating that girls show significantly higher levels of math anxiety as compared to boys [61,76,77].

As expected, third graders had a higher performance on the mathematical achievement standardized test as compared to second graders and especially in subtraction, multiplication, and division. These results are consistent with our previous findings, as these mathematical skills are affected most by working memory, which expands during childhood [78]. It is important to note that third graders scored significantly better than second graders on inductive reasoning geometric tasks.

Finally, based on the results, as well as on the results of its structural validity and reliability, we can conclude that mathematical achievement test seems to be a useful and reliable scale for the assessment of second and third graders. It could be the base for the formation of an assessment tool, which will take into account the cognitive aspects of mathematical knowledge, in order to detect with accuracy primary school students with MD. The updated version of the mathematical achievement test will incorporate new tasks, except those based on the corresponding curriculum, which will target the assessment of students’ sustained attention, inductive reasoning, and working memory.

To summarize, with the current study, we attempted to evaluate the cognitive factors of mathematical achievement in second- and third-grade students in Greek primary schools. Results of the present study are consistent with recent findings, verifying that sustained attention, inductive reasoning, working memory, and math anxiety are correlated with young students’ mathematical performance. In addition, according to the results, mental arithmetic ability, sustained attention, and working memory can predict math achievement. Finally, the results of the study did not indicate gander-based difference on math achievement. However, given that this is a pilot study and the sample is limited, these results cannot be generalized to a larger population. Another limitation of the study is that construct validity testing was not conducted for the translated version of the math anxiety scale.

## Figures and Tables

**Table 1:** Correlation matrix (two-tailed) for mathematical measures, cognitive skills, and math anxiety.

	1	1.1	1.2	1.3	1.4	1.5	1.6	1.7	1.8	2	3	4	5	6	7
1. Math Achievement	1														
*1.1 Counting*	0.242 *	1													
*1.2 Addition*	0.626 **	0.090	1												
*1.3 Subtraction*	0.701 **	−0.065	0.511 **	1											
*1.4 Multiplication*	0.812 **	0.186	0.358 **	0.482 **	1										
*1.5 Division*	0.821 **	0.157	0.397 **	0.523 **	0.702 **	1									
*1.6 Patterns*	0.593 **	0.167	0.301 **	0.285 **	0.422 **	0.377 **	1								
*1.7 Number Line*	0.483 **	0.005	0.243 *	0.247 *	0.269 *	0.362 **	0.161	1							
*1.8 Problem Solving*	0.635 **	0.100	0.324 **	0.259 *	0.422 **	0.345 **	0.480 **	0.221 *	1						
2. Sustained Attention	0.368 **	0.167	0.252 *	0.196	0.357 **	0.315 **	0.158	0.291 **	0.125	1					
3. Inductive Reasoning (Stories)	0.041	0.031	−0.046	−0.006	0.094	0.065	0.108	0.007	−0.043	0.076	1				
4. Inductive Reasoning (Geometry)	0.313 **	−0.040	0.082	0.200	0.351 **	0.309 **	0.292 **	0.139	0.129	0.265 *	0.303 **	1			
5. Mental Arithmetic	0.542 **	0.203	0.274 **	0.251 *	0.442 **	0.486 **	0.414 **	0.375 **	0.306 **	0.142	0.248 *	0.305 **	1		
6. Math Anxiety	−0.263 *	0.091	−0.044	−0.205	−0.186	−0.255 *	−0.238 *	−0.237 *	−0.165	−0.083	−0.155	−0.212 *	−0.299 **	1	
7. Working Memory	−0.420 **	−0.106	−0.257 *	−0.283 **	−0.286 **	−0.410 **	−0.233 *	−0.204	−0.267 *	−0.094	−0.093	−0.314 **	−0.386 **	0.079	1

* Correlation is significant at the 0.05 level, ** Correlation is significant at the 0.01 level.

**Table 2:** Multiple regression analysis predicting mathematical achievement.

	*R* ^2^	$\mathrm{Adjusted}\;R^{2}$	$R^{2}\mathrm{Change}$	F Change
**Model 1** Mental Arithmetic	0.292	0.284	0.292	36.316 ***
**Model 2** Mental Arithmetic Sustained Attention	0.378	0.364	0.086	12.023 ***
**Model 3** Mental Arithmetic Sustained Attention Working Memory	0.424	0.404	0.046	6.813 *

* Significant at the 0.05 level, ** Significant at the 0.01 level, *** Significant at the 0.001 level.

**Table 3:** Descriptive statistics for mathematical measures and cognitive skills in terms of the school grade. *

	Year 2 (N = 53)		Year 3 (N = 38)	
Measures	M	SD	M	SD
Mathematical Skills	22.38 (32)	5.80	27.68	4.11
Counting	3.77 (4)	0.64	3.63	0.67
Addition	2.91 (4)	0.96	3.66	0.48
Subtraction	1.62 (4)	1.24	3.05	1.11
Multiplication	2.02 (4)	1.39	3.34	1.14
Division	2.30 (4)	1.55	3.24	1.28
Patterns	3.57 (4)	0.84	3.87	0.41
Number Line	3.38 (4)	0.90	3.61	0.87
Problem Solving	2.83 (4)	1.40	3.29	1.09
Sustained Attention	0.24	0.03	0.26	0.04
Inductive Reasoning (Stories)	4.02 (5)	1.07	4.05	0.93
Inductive Reasoning (Geometry)	8.08 (12)	2.81	9.63	2.07
Mental Arithmetic	11.06 (30)	2.90	11.79	2.89
Math Anxiety	20.89	5.54	18.24	6.02
Working Memory	8.96	12.90	5.61	9.19

* Maximum score for each measure is shown in parenthesis. The optimal score for math anxiety and working memory is 0.

**Table 4:** Descriptive statistics for mathematical measures and cognitive skills in terms of gender.

	Male (N = 42)		Female (N = 49)	
Measures	M	SD	M	SD
Mathematical Skills	25.55	5.60	23.78	5.85
Counting	3.71	0.55	3.71	0.74
Addition	3.14	1.00	3.29	0.79
Subtraction	2.48	1.37	2.00	1.37
Multiplication	2.81	1.44	2.37	1.44
Division	2.79	1.49	2.61	1.54
Patterns	3.95	0.31	3.47	0.87
Number Line	3.33	1.10	3.59	0.67
Problem Solving	3.36	1.01	2.73	1.44
Sustained Attention	0.24	0.41	0.251	0.035
Inductive Reasoning (Stories)	4.07	1.03	4.00	1.00
Inductive Reasoning (Geometry)	9.15	2.25	8.35	2.90
Mental Arithmetic	12.00	3.11	10.86	2.66
Math Anxiety	18.88	6.18	20.55	5.52
Working Memory	8.19	10.83	7.02	12.25
